# Synergistic antitumor activity of oncolytic reovirus and chemotherapeutic agents in non-small cell lung cancer cells

**DOI:** 10.1186/1476-4598-8-47

**Published:** 2009-07-14

**Authors:** Shizuko Sei, Jodie K Mussio, Quan-en Yang, Kunio Nagashima, Ralph E Parchment, Matthew C Coffey, Robert H Shoemaker, Joseph E Tomaszewski

**Affiliations:** 1 Laboratory of Human Toxicology and Pharmacology, SAIC-Frederick, Inc, NCI-Frederick, Frederick, Maryland, USA; 2 Electron Microscope Laboratory, SAIC-Frederick, Inc, NCI-Frederick, Frederick, Maryland, USA; 3 Oncolytics Biotech Inc, Calgary, Alberta, Canada; 4 Developmental Therapeutics Program, National Cancer Institute, Frederick, Maryland, USA; 5 Division of Cancer Treatment and Diagnostics, National Cancer Institute, Bethesda, Maryland, USA

## Abstract

**Background:**

Reovirus type 3 Dearing strain (ReoT3D) has an inherent propensity to preferentially infect and destroy cancer cells. The oncolytic activity of ReoT3D as a single agent has been demonstrated *in vitro *and *in vivo *against various cancers, including colon, pancreatic, ovarian and breast cancers. Its human safety and potential efficacy are currently being investigated in early clinical trials. In this study, we investigated the *in vitro *combination effects of ReoT3D and chemotherapeutic agents against human non-small cell lung cancer (NSCLC).

**Results:**

ReoT3D alone exerted significant cytolytic activity in 7 of 9 NSCLC cell lines examined, with the 50% effective dose, defined as the initial virus dose to achieve 50% cell killing after 48 hours of infection, ranging from 1.46 ± 0.12 ~2.68 ± 0.25 (mean ± SD) log_10 _pfu/cell. Chou-Talalay analysis of the combination of ReoT3D with cisplatin, gemcitabine, or vinblastine demonstrated strong synergistic effects on cell killing, but only in cell lines that were sensitive to these compounds. In contrast, the combination of ReoT3D and paclitaxel was invariably synergistic in all cell lines tested, regardless of their levels of sensitivity to either agent. Treatment of NSCLC cell lines with the ReoT3D-paclitaxel combination resulted in increased poly (ADP-ribose) polymerase cleavage and caspase activity compared to single therapy, indicating enhanced apoptosis induction in dually treated NSCLC cells. NSCLC cells treated with the ReoT3D-paclitaxel combination showed increased proportions of mitotic and apoptotic cells, and a more pronounced level of caspase-3 activation was demonstrated in mitotically arrested cells.

**Conclusion:**

These data suggest that the oncolytic activity of ReoT3D can be potentiated by taxanes and other chemotherapeutic agents, and that the ReoT3D-taxane combination most effectively achieves synergy through accelerated apoptosis triggered by prolonged mitotic arrest.

## Background

The use of viral vectors for cancer gene therapy has been vigorously explored for the last two decades. The overall goal of the strategy is to promote cancer cell death through various means, such as tumor suppressor gene replacement, oncogene inactivation, suicide gene delivery, drug sensitization or enhancement of anticancer immunity. The extensive research efforts to develop tumor cell death-inducing viral vectors have reignited the interest in oncolytic viruses in recent years as a promising group of viral therapeutics that can directly induce tumor cell lysis through viral replication. The latest multidisciplinary research in cancer genomics and proteomics further provides an opportunity to discern various molecular pathways specifically upregulated (or dysregulated) in cancers that can be exploited as part of viral replication and destruction machinery. Indeed, many replication-competent oncolytic viruses currently in development are recombinant viruses engineered to become reliant on such cancer-specific molecules and signaling pathways for viral entry and replication, thus rendering cancer cells more selectively susceptible to virus-mediated oncolysis [1]. Unlike chemical entity-based anticancer agents, these viruses can propagate in susceptible tumor cells, re-target, infect, and destroy remaining cancer cells within the primary tumor or in the metastases, repeating the cycle until viral spread is halted by the host antiviral response or by mechanical barriers such as loss of vasculature and necrotic tissues.

Mammalian reoviruses are ubiquitous, non-enveloped dsRNA viruses, normally associated with relatively benign pathology in humans. The Dearing strain of reovirus serotype 3 (ReoT3D) is a non-engineered wild type reoviral strain and belongs to a growing number of the new generation of oncolytic viruses because of its innate ability to preferentially kill transformed cells [2,3]. The oncolytic potency of ReoT3D has been extensively demonstrated against various cancers *in vitro *and *in vivo*, including colon, pancreatic, ovarian and breast cancers, as well as malignant gliomas and lymphoid malignancies [4-9]. The safety, feasibility and potential efficacy of ReoT3D cancer therapy are currently being investigated in phase I/II clinical trials [10]. As with other emerging therapeutics for cancer, the combined regimen of ReoT3D and conventional chemotherapeutic agents is expected to play a significant role in future clinical applications. However, it is currently unknown whether conventional chemotherapeutic agents can augment or interfere with the oncolytic effect of ReoT3D. In this study, we evaluated the oncolytic activity of ReoT3D in non-small cell lung cancer (NSCLC), and explored the therapeutic feasibility of ReoT3D-chemotherapeutic combination regimens against NSCLC.

## Results

### Oncolytic activity of ReoT3D and progeny virion production in NSCLC cell lines

**Figure 1:**
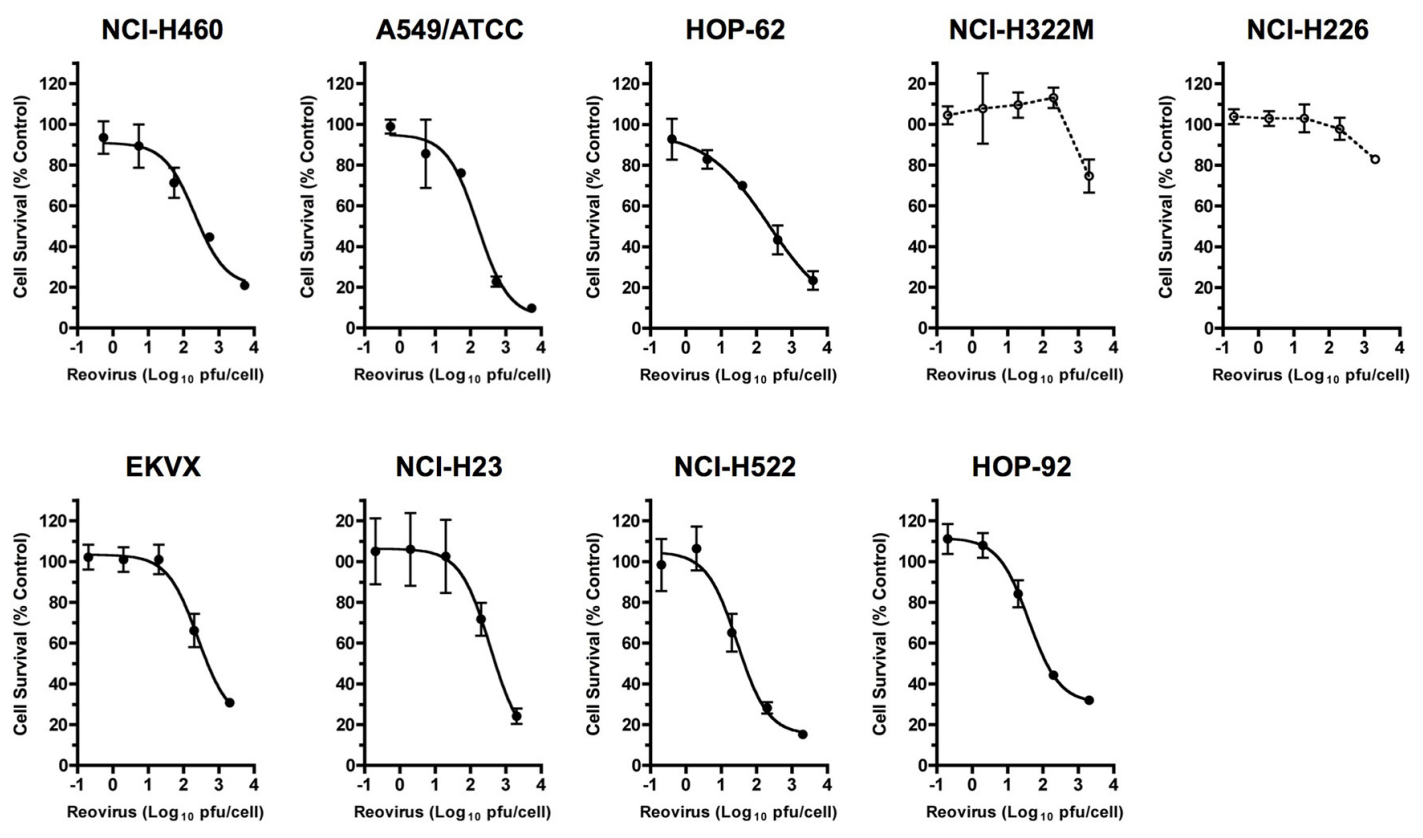
**Oncolytic effect of reovirus type 3 Dearing strain (ReoT3D) in non-small cell lung cancer (NSCLC) cells**. Dose-dependent cell death shown as % cell survival (mean ± SD) induced by ReoT3D in 9 NSCLC cell lines evaluated by the XTT assay [49] at 48 hours post-infection. ReoT3D dose is defined as multiplicity of infection (MOI) expressed in plaque forming units per cell (pfu/cell). The curves were fitted by non-linear regression using GraphPad Prism (GraphPad Software, Inc., San Diego) for 7 permissive cell lines, NCI-H460, A549/ATCC, HOP-62, EKVX, NCI-H23, NCI-H522, and HOP-92. The data shown are representative of 3 separate experiments.

**Figure 2:**
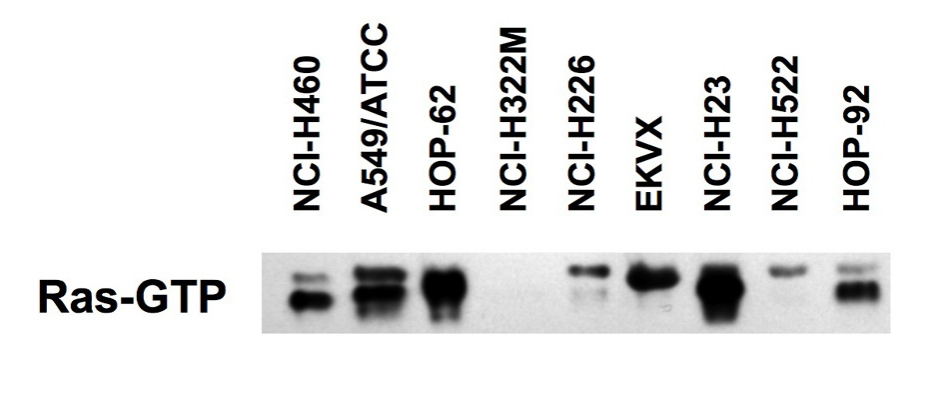
**Levels of GTP-bound activated Ras protein in 9 NSCLC cell lines analyzed by Western blot, using anti-pan-Ras antibody**. Note, high levels of activated Ras were observed with A549/ATCC (K-*ras*^G12S^) [13], HOP-62 (K-*ras*^G12C^) [13], and NCI-H23 (K-*ras*^G12C^) [13], followed by NCI-H460 (K-*ras*^Q61H^) [13], EKVX and HOP-92. The levels of activated Ras were significantly lower not only with ReoT3D-resistant NCI-H322M and NCI-H226, but also with ReoT3D sensitive cell line, NCI-H522. Also see table 1 for comparison of Ras-activating gene mutation status and ReoT3D sensitivity of each cell line.

We first examined the *in vitro *susceptibility of human NSCLC cell lines to ReoT3D, as reoviral oncolytic activity had not been extensively studied in human lung cancer cells. Nine NSCLC cell lines (NCI-H460, A549/ATCC, HOP-62, NCIH322M, NCI-H226, EKVX, NCI-H23, NCI-H522, and HOP-92) included in the NCI-60 tumor cell line panel (Developmental Therapeutics Program, DTP, NCI-Frederick, Frederick, MD) were incubated with serially diluted ReoT3D for cytopathic effect (CPE) determination. Within 48 hours post-infection, ReoT3D induced significant cell death in seven of nine NSCLC cell lines in a dose-dependent manner (Figure 1). ReoT3D 50 percent effective dose (ED50), defined as the initial virus dose (multiplicity of infection, MOI, expressed in plaque forming units per cell, pfu/cell) that resulted in 50% cell viability at 48 hours post-inoculation as compared to untreated controls, ranged from 1.46 ± 0.12 to 2.68 ± 0.25 (mean ± SD from 3 separate experiments) log_10 _pfu/cell in the sensitive cell lines (Table 1). In contrast, NCI-H226 and NCI-H322M were relatively resistant to ReoT3D in this short-term incubation assay, as indicated by the significantly lower levels of cell death even at the highest inoculum dose compared to those seen in the sensitive cell lines (*P *< 0.0001) (Figure 1). While ReoT3D has been shown to induce CPE in murine cells with an activated Ras pathway [11,12], the presence of Ras-activating gene mutations [13] or activated Ras was not necessarily associated with lower ReoT3D ED50 in the NSCLC cell lines tested in our study (Table 1 and Figure 2).

**Table 1:** Oncolytic activity of ReoT3D in 9 NSCLC cell lines included in the NCI-60 cell line panel

**NSCLC Cell Line**	**ED50 (log_10 _pfu/cell: mean ± SD)**
NCI-H460*	2.68 ± 0.25
A549/ATCC*	2.51 ± 0.27
HOP-62*	2.56 ± 0.19
NCI-H322M	> 3.30
NCI-H226	> 3.30
EKVX	2.47 ± 0.34
NCI-H23*	2.47 ± 0.15
NCI-H522	1.46 ± 0.12
HOP-92	1.96 ± 0.27

ED50: 50% effective dose defined by the initial MOI (pfu/cell) to achieve 50% cell death in 48 hours after infection. Shown are mean ± SD from 3 independent experiments. *Asterisk denotes cell lines with *ras *gene mutation. Abbreviations: ReoT3D, reovirus type 3 Dearing; NSCLC, non-small cell lung cancer; MOI, multiplicity of infection; pfu, plaque forming units.

### *In vitro *combination effects of ReoT3D and chemotherapeutic agents against NSCLC cells

**Figure 3:**
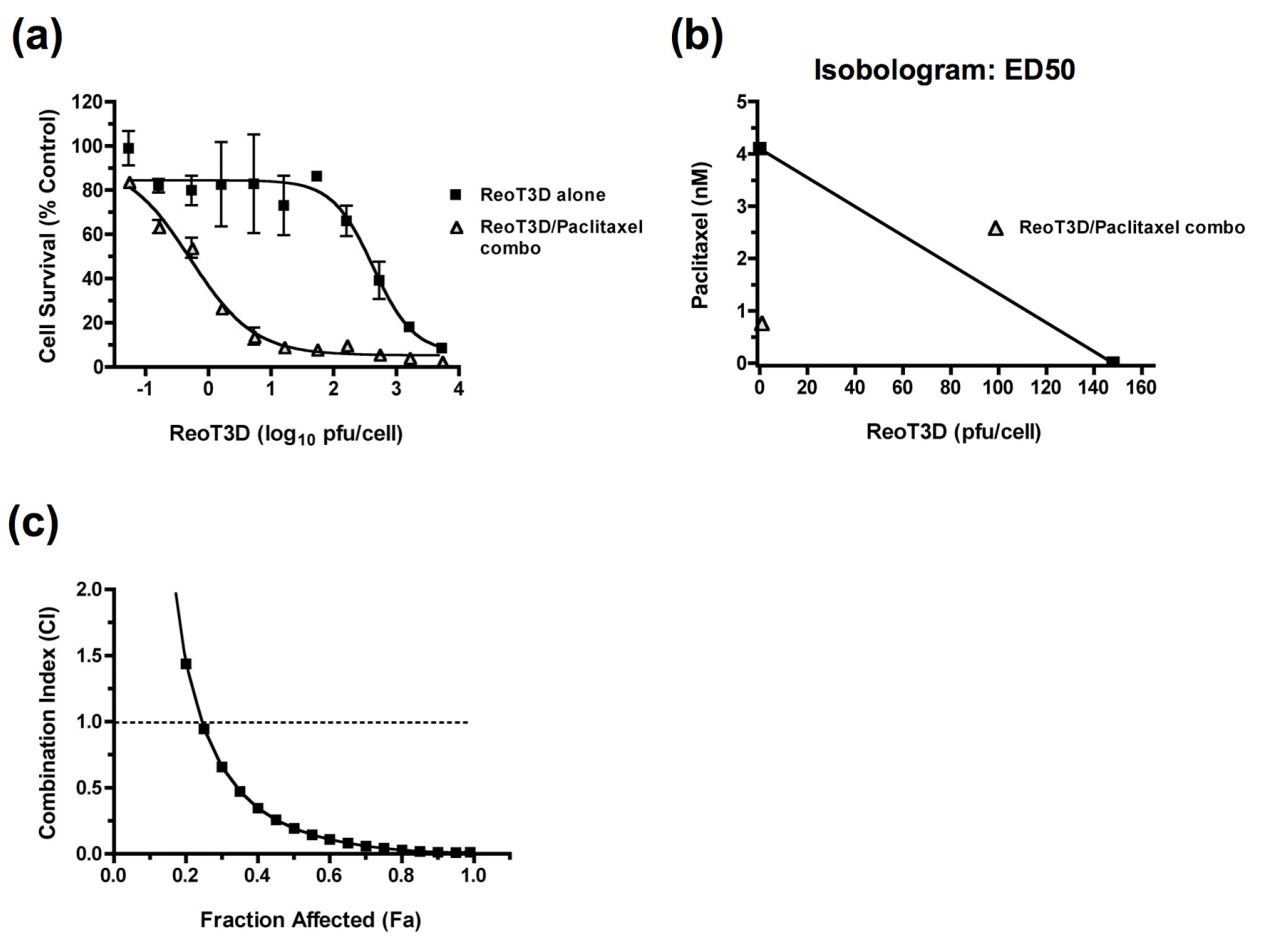
**Synergistic activity of ReoT3D and paclitaxel combination in NCI-H460 cells** as demonstrated by (a) the leftward shift of the dose response curve (error bars: SD), (b) isobologram analysis (shown are for the ED50 doses), and (c) fractional effect-combination index (CI) plot generated by CalcuSyn (Biosoft, Ferguson, MO). The data shown are representative of 3 separate experiments.

Next, we investigated the combination effects of ReoT3D and chemotherapeutic agents in four ReoT3D-susceptible (NCI-H460, HOP-92, NCIH23, and EKVX) and two relatively resistant (NCI-H226 and NCI-H322M) cell lines using the Chou-Talalay method. Growth inhibitory effects of chemotherapeutic agents, paclitaxel, cisplatin, gemcitabine, and vinblastine, were first determined in each cell line by the XTT cytotoxicity assay and expressed as the drug concentration that inhibited cell growth by 50% compared to untreated control (IC50) (Table 2). Of the 6 cell lines, NCI-H322M and EKVX showed high levels of resistance to multiple compounds, consistent with the previous report that characterized them as multi-drug resistant against a variety of anticancer agents [14] (Table 2). When NCI-H460 cells were incubated with increasing doses of ReoT3D in the absence or presence of paclitaxel, whose concentration was serially increased to maintain a constant ratio to the ReoT3D dosage (the constant ratio combination design) [15], the synergistic anticancer effect of the two agents was clearly demonstrated by a leftward shift of the dose response curve (Figure 3a) as well as isobologram and combination index (CI) analyses [16,17] (Figure 3b and 3c, respectively). The combination effects of ReoT3D and other chemotherapeutic agents were similarly examined in all 6 cell lines using at least two different dosage ratios, and CI values were obtained for each combination regimen (Table 2). In many instances, the combination of ReoT3D with different chemotherapeutic agents was found to exert moderate to strong synergistic effects at both combination ratios as shown by the CI that were consistently smaller than 1.0 at ED50, ED75 and ED90 (Table 2). Interestingly, with the exception of ReoT3D-paclitaxel combination, antagonistic effects (CI > 1.0) of ReoT3D-chemotherapeutic combinations were typically observed in NSCLC cell lines that exhibited high-level resistance to the test compounds with IC50 often exceeding the highest test concentration, 100 μM (Table 2). In contrast, the combination of ReoT3D and paclitaxel was consistently synergistic in all the NSCLC cell lines tested, regardless of the level of paclitaxel sensitivity (Table 2). The relative resistance of NSCLC cell lines to ReoT3D did not appear to negatively influence the outcomes, as the synergistic effect was clearly seen in NCI-H226 for all the combination regimens examined (Table 2).

**Table 2:** Combination index values for ReoT3D-chemotherapeutic combination regimens in NSCLC cells

			**Combination Index Values^§^**					**Combination Index Values^§^**			
**Drugs & Cell Lines**	**Drug Sensitivity^¶ ^IC50 (μM)**	Ratio* I	**ED50**	**ED75**	**ED90**	**Mean**	Ratio* II	**ED50**	**ED75**	**ED90**	**Mean**
Paclitaxel											
NCI-H460	0.003 ± 0.001	1:53	0.903	0.221	0.057	0.394	1:533	0.193	0.042	0.013	0.083
HOP-92	38.50 ± 16.01	1:20	0.144	0.295	0.613	0.351	1:200	0.178	0.420	0.994	0.530
NCI-H23	0.08 ± 0.04	1:20	0.577	0.303	0.159	0.347	1:200	0.329	0.061	0.011	0.134
EKVX	56.06 ± 15.87	1:20	0.294	0.440	0.660	0.464	1:200	0.124	0.216	0.374	0.238
NCI-H226	77.79 ± 25.08	1:20	0.543	0.344	0.218	0.368	1:200	0.004	1.30E-04	4.73E-06	0.001
NCI-H322M	> 100	1:20	0.069	0.092	0.133	0.098	1:200	0.078	0.081	0.084	0.081
Cisplatin											
NCI-H460	5.31 ± 1.21	1:53	0.875	0.313	0.112	0.433	1:533	0.661	0.292	0.129	0.361
HOP-92	6.82 ± 3.22	1:20	0.352	0.325	0.500	0.392	1:200	0.053	0.080	0.195	0.109
NCI-H23	3.51 ± 1.15	1:20	0.579	0.487	0.530	0.532	1:200	0.546	0.248	0.342	0.379
EKVX	29.34 ± 6.62	1:20	0.955	0.559	0.328	0.614	1:200	0.933	0.754	0.610	0.766
NCI-H226	14.71 ± 6.60	1:20	0.615	0.297	0.145	0.352	1:200	0.558	0.081	0.013	0.217
NCI-H322M	> 100	1:20	> 10	> 10	> 10	> 10	1:200	> 10	> 10	> 10	> 10
Gemcitabine											
NCI-H460	0.01 ± 0.003	1:53	0.199	0.126	0.126	0.150	1:533	0.006	0.017	0.054	0.025
HOP-92	0.83 ± 0.61	1:20	0.372	0.117	0.047	0.179	1:200	0.099	0.110	0.127	0.112
NCI-H23	0.02 ± 0.01	1:20	0.630	0.249	0.285	0.388	1:200	0.207	0.092	0.369	0.223
EKVX	> 100	1:20	> 10	> 10	> 10	> 10	1:200	> 10	> 10	> 10	> 10
NCI-H226	17.69 ± 10.16	1:20	0.112	0.030	0.008	0.050	1:200	0.057	0.007	0.001	0.021
NCI-H322M	> 100	1:20	> 10	> 10	> 10	> 10	1:200	> 10	> 10	> 10	> 10
Vinblastine											
NCI-H460	0.92 ± 0.35 (nM)	1:5.3	0.255	0.149	0.089	0.164	1:53.3	0.069	0.045	0.031	0.049
HOP-92	48.80 ± 23.08	1:20	1.749	7.049	> 10	> 10	1:200	0.133	1.256	11.922	4.437
NCI-H23	1.49 ± 1.09 (nM)	1:2	0.316	0.056	0.019	0.130	1:20	0.367	0.052	0.008	0.142
EKVX	> 100	1:20	3.275	1.927	1.134	2.112	1:200	0.193	0.651	2.192	1.012
NCI-H226	28.46 ± 14.13	1:20	0.081	0.043	0.023	0.049	1:200	0.027	0.006	0.001	0.012
NCI-H322M	> 100	1:20	> 10	> 10	> 10	> 10	1:200	> 10	> 10	> 10	> 10

^¶^Sensitivities of 6 NSCLC cell lines to each listed drug tested as a single agent (mean ± SD from 3 separate experiments). *Constant ratios of drug concentrations in μM (or nM) and ReoT3D MOI doses in plaque forming units per cell used for combination studies (see text). ^§^Combination index (CI) values shown are from representative experiments repeated three times with similar results. Abbreviations: ReoT3D, reovirus type 3 Dearing; NSCLC, non-small cell lung cancer; IC50, 50% inhibitory concentration; MOI, multiplicity of infection expressed in plaque forming units per cell.

These data demonstrated that while the oncolytic activity of ReoT3D could be generally potentiated by the chemotherapeutic agents that alone were cytotoxic to the tested cell lines, paclitaxel appeared to exert unique effects on the cell-death process induced by ReoT3D, even in the cells with reduced sensitivity to the compound.

### Progeny virion production from NSCLC cells treated with the combination of ReoT3D and chemotherapeutic agents

**Figure 4:**
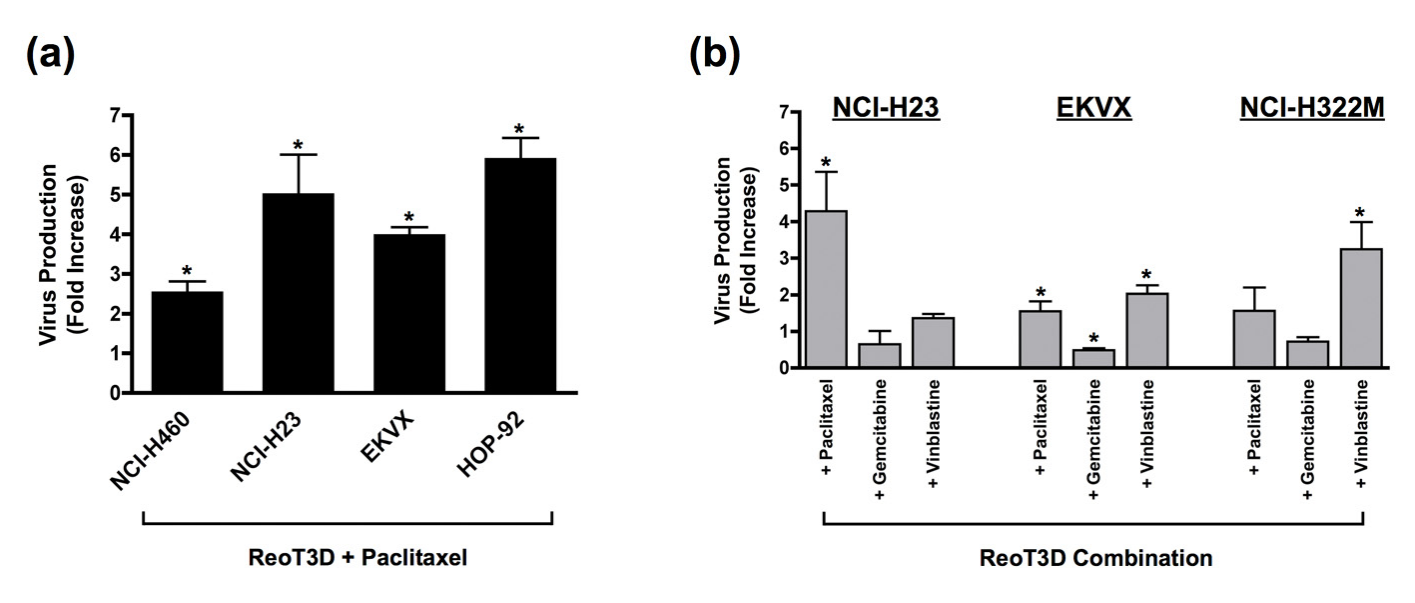
**(a) Fold increases (mean ± SD) in infectious progeny virion production** determined by plaque assay in NCI-H460, NCI-H23, EKVX and HOP-92 treated with the combination of ReoT3D (MOI = 20) and paclitaxel (1 μM for NCI-H460 and NCI-H23 and 10 μM for EKVX and HOP-92 cells) for 24 hours as compared to ReoT3D alone (MOI = 20). Paclitaxel invariably increased the level of virion production from all the cell lines tested. **(b) **Fold increases (mean ± SD) in infectious progeny virion production in NCI-H23, EKVX and NCI-H322M treated with ReoT3D (MOI = 20) in combination with paclitaxel (0.1 μM for NCI-H23 and 1 μM for EKVX and NCI-H322M), gemcitabine (0.1 μM for NCI-H23 and 1 μM for EKVX and NCI-H322M) or vinblastine (10 nM for NCI-H23 and 100 nM for EKVX and NCI-H322M) for 24 hours as compared to ReoT3D alone (MOI = 20). Note the increased virion production in the presence of tubulin-binding agents, paclitaxel or vinblastine, but not with gemcitabine. Asterisks denote statistical significance (*P *< 0.05).

Next, we asked whether the synergistic oncolytic effects of ReoT3D-chemotherapeutic combination regimens reflected increases in virally induced lytic cell death, driven by a burst of progeny virion production from the cells. To capture early changes in viral production levels, the amounts of infectious virions released into the culture supernatants in the first 24 hours were compared among NSCLC cell lines infected with ReoT3D in the absence or presence of different chemotherapeutic agents. Notably, the addition of paclitaxel to ReoT3D (MOI = 20) was found to increase the level of progeny virion production as compared to virus alone in all 4 cell lines tested, NCI-H460, NCI-H23, EKVX and HOP-92 (*P *= 0.001, 0.03, 0.003 and 0.0001, respectively) (Figure 4a). However, such augmentation of virion production was not unique to paclitaxel nor associated with synergy, as ReoT3D-vinblastine combination also significantly increased the level of progeny virions released from EKVX and NCI-H322M (*P *< 0.01) (Figure 4b), where the combination was not associated with a synergistic effect (mean CI > 1.0). In contrast to paclitaxel and vinblastine, the addition of gemcitabine was not associated with an increased reoviral progeny virion production in NSCLC cells (Figure 4b). These data suggested that the synergistic effects of ReoT3D-chemotherapeutic combinations did not directly result from lytic necrosis induced by rapid increases in virion release, but probably stemmed from certain changes in programmed cell death pathways.

### PARP cleavage in NSCLC cells treated with ReoT3D-chemotherapeutic combination regimens

**Figure 5:**
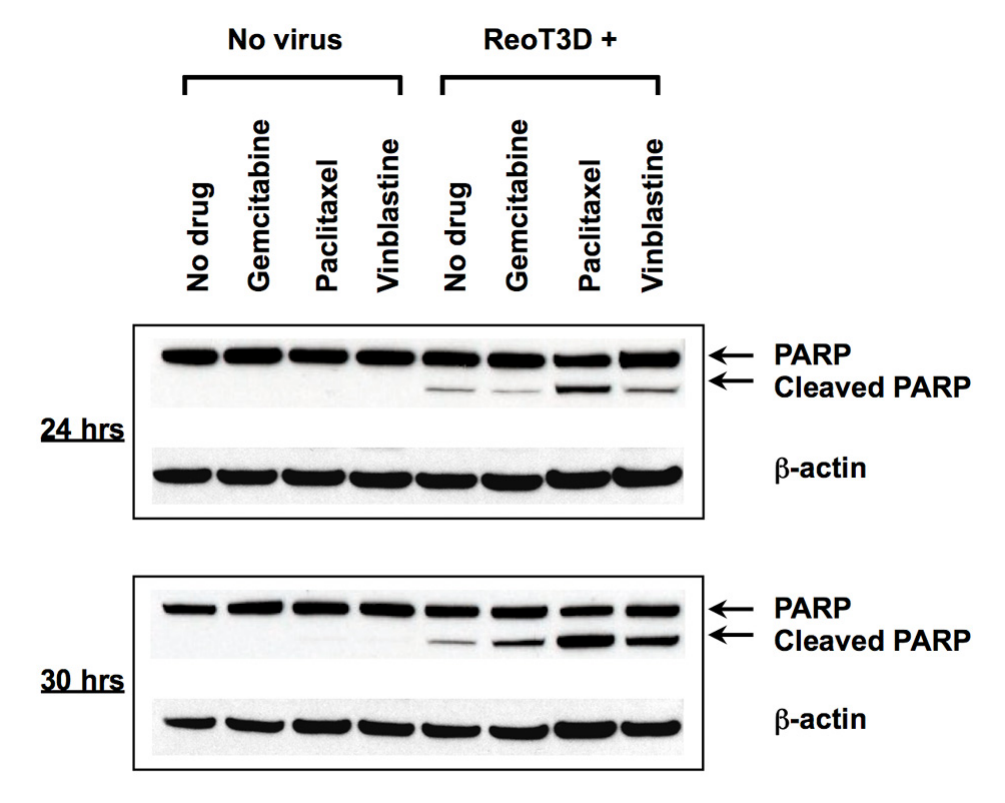
**Western blot analysis of poly (ADP-ribose) polymerase (PARP) cleavage** in cell lysates obtained from NCI-H460 cultured for 24 to 30 hrs after infection with ReoT3D (MOI = 20) in the absence or presence of gemcitabine (1 μM), paclitaxel (1 μM) or vinblastine (0.1 μM). Shown are the full-length PARP (116 kDa) and the larger fragment (89 kDa) of apoptotically cleaved products, as well as β-actin used as a loading control. Results are representative of 2 separate experiments.

Reovirus type 3 Abney (ReoT3A) has been shown to induce apoptotic cell death in various cancer cell lines, including in 2 NSCLC cell lines, NCI-H157 and A549 [18]. To further investigate the mechanistic basis for the synergistic activity of ReoT3D-chemotherapeutic combinations in NSCLC cells, we examined the proteolytic cleavage of poly (ADP-ribose) polymerase (PARP) in NCI-H460 treated with 20 MOI of ReoT3D alone or in combination with gemcitabine, paclitaxel or vinblastine for 24 to 30 hours. Western blot analysis of cell lysates demonstrated the cleaved 89-kDa PARP fragment in ReoT3D-treated NCI-H460, whereas treatment with gemicitabine (1 μM), paclitaxel (1 μM) or vinblastine (0.1 μM) alone resulted in virtually undetectable PARP cleavage in this cell line (Figure 5). Cells treated with the combination of ReoT3D and paclitaxel showed significantly higher levels of PARP cleavage than ReoT3D alone at 24 and 30 hours post-treatment, accompanied by a gradual decrease in the amount of full-length PARP (Figure 5). Increased PARP cleavage was also observed with the combination of ReoT3D and other chemotherapeutic agents at 24 or 30 hours post-treatment, although to a lesser extent than ReoT3D-paclitaxel combination (Figure 5). These data suggested that the synergistic cell death induced by the ReoT3D-paclitaxel combination was mediated at least in part by the enhanced apoptotic process in NCI-H460.

### Activation of caspases in NSCLC cells treated with ReoT3D alone or in combination with paclitaxel

**Figure 6:**
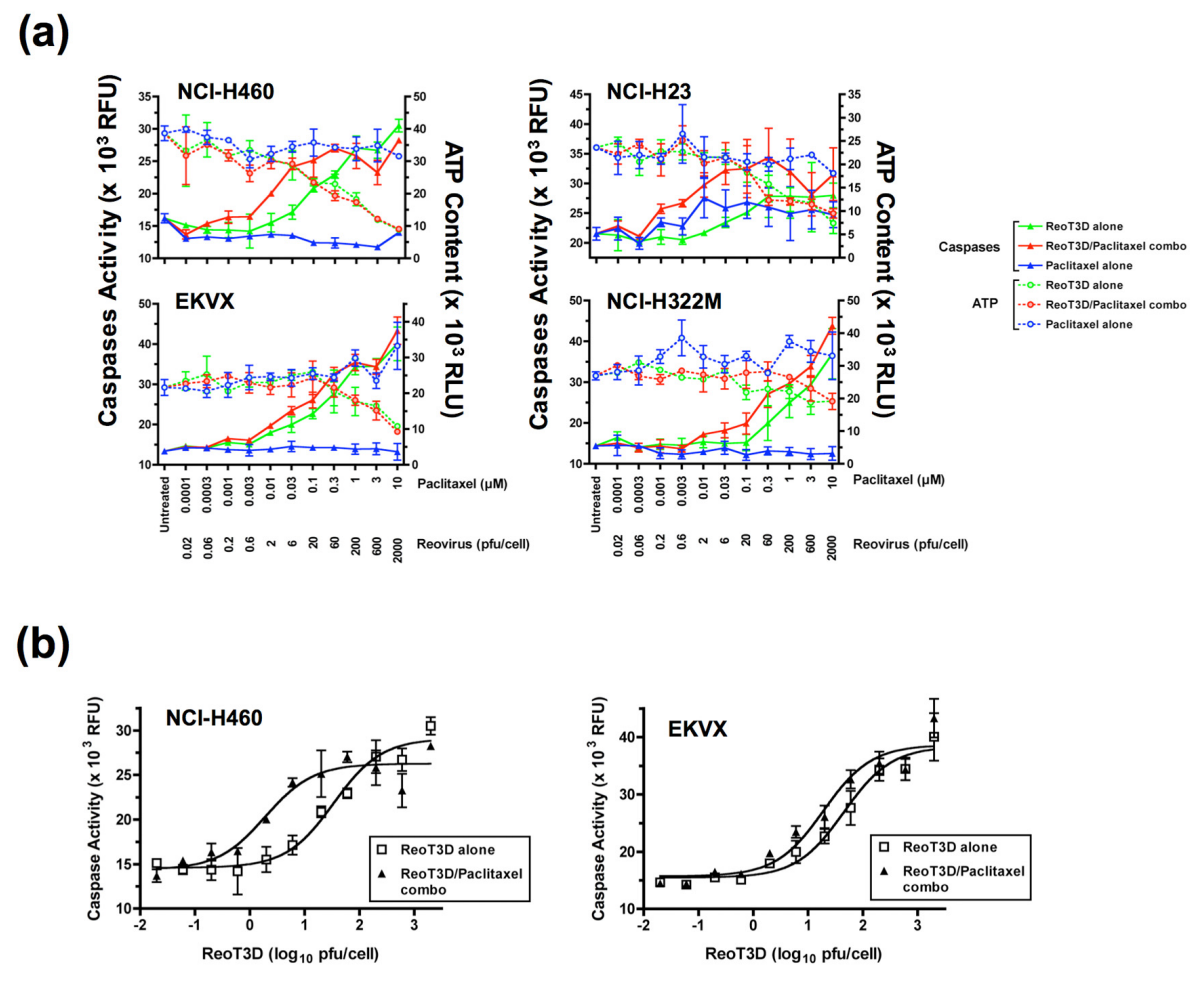
**(a) Levels of caspase activity and ATP content in NCI-H460, NCI-H23, EKVX and NCI-H322M cells** treated with increasing doses of either ReoT3D or paclitaxel alone, or both in combination for 24 hours, using the constant ratio combination design [15]. This design format was employed to evaluate the synergistic activity of ReoT3D-chemotherapeutic combination regimens (see text). Shown are the mean ± SD in relative fluorescence units (RFU) and relative luminescence units (RLU) for caspase activity (closed triangle) and ATP content (open circle), respectively, for ReoT3D alone (green), ReoT3D-paclitaxel combination (red) and paclitaxel alone (blue). The data shown are representative of 3 separate experiments. **(b) Dose-response curves of caspase activation** induced by treatment with ReoT3D alone or ReoT3D-paclitaxel combination for 24 hours fitted by non-linear regression using GraphPad Prism (GraphPad Software, Inc.). Shown as an example are non-linear regression analyses for NCI-H460 and EKVX. See text for statistical analysis on the best-fit values.

To explore whether the enhancement of apoptosis played a role in the synergistic activity of the ReoT3D-paclitaxel combination in other NSCLC cell lines, we examined the level of caspase activity in NCI-H460, NCI-H23, EKVX and NCI-H322M treated with increasing doses of either ReoT3D or paclitaxel alone or both in combination, using the constant ratio combination design [15], the same dosing scheme adopted for CI analyses of Chou-Talalay (see above). In each of 4 cell lines, treatment with ReoT3D alone led to a significant increase in caspase activity in a dose-dependent manner, although the response appeared less robust in NCI-H322M, where higher ReoT3D MOIs were required to achieve similar magnitudes of caspase activation above baseline than in 3 other cell lines (Figure 6a). The increases in caspase activity were associated with dose-dependent decreases in cell viability as assessed by intracellular ATP content in ReoT3D-treated cells (Figure 6a). Paclitaxel alone had virtually no effects on caspase activation or ATP content during the 24-hr exposure, except in NCI-H23 that showed moderate increases in caspase activity upon treatment with the compound at ≥ 10 nM (Figure 6a). When ReoT3D was combined with paclitaxel, the levels of caspase activation appeared more pronounced as compared to ReoT3D single therapy, as seen by a leftward shift of the dose response curves (Figure 6a). Similar results were obtained from 3 separate experiments for all 4 cell lines.

In order to evaluate whether the extent of caspase activation induced by the ReoT3D-paclitaxel combination was significantly different from ReoT3D single treatment in these cell lines, the caspase activity dose-response curves were fitted by non-linear regression using GraphPad Prism (GraphPad Software Inc., San Diego, CA) (Figure 6b). The best-fit values of a variable, LogEC50, obtained by the Prism analysis from 3 independent experiments were then compared between the two treatment regimens, ReoT3D alone vs. ReoT3D-paclitaxel combination, using a paired t-test. The analysis demonstrated that the activation of caspases was significantly enhanced with the ReoT3D-paclitaxel combination therapy as compared to ReoT3D alone in NCI-H460, NCI-H23, EKVX and NCIH322M (*P *= 0.004, 0.03, 0.04, and 0.02, respectively). These data suggested that enhanced apoptotic cell death most likely constituted the synergistic cell killing induced by the combination of ReoT3D and paclitaxel in NSCLC cells.

### Dynamic effects of ReoT3D and paclitaxel on cell cycle progression and caspase activation

ReoT3D infection has been shown to induce cell cycle arrest at G1/S and G2/M [19,20]. Antimicrotubule agents, including taxanes and vinca alkaloids, activate the spindle-assembly checkpoint and induce mitotic arrest at the metaphase and anaphase transition, which ultimately leads to cell death by apoptosis [21]. A certain proportion of cells exposed to these antimitotic agents may undergo an aberrant mitotic exit without cytokinesis, forming multinucleated cells in interphase [21]. To gain mechanistic insight into the enhanced apoptotic cell death induced by the ReoT3D-paclitaxel combination, we investigated the effects of ReoT3D and paclitaxel on cell cycle and caspase-3 activation in NSCLC cells using flow cytometry.

**Figure 7:**
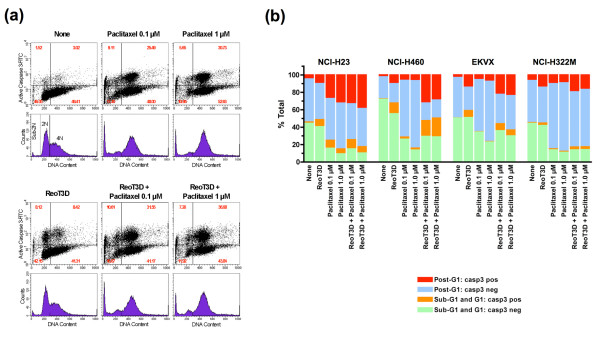
**Flow cytometric analysis of DNA content and caspase-3 activation in NSCLC cells treated with either ReoT3D (MOI = 20) or paclitaxel alone (0.1 or 1 μM), or both in combination**. (a) Shown are scattergrams (top) and histograms (bottom), ungated, of NCI-H23 cells treated with the indicated agent(s) for 24 hours. Harvested cells were fixed, permeabilized and stained with FITC-conjugated anti-active caspase-3 antibody followed by PI staining. Each histogram depicts the DNA content of cells in G1 (2N) and G2/M (4N), while the scattergrams demonstrate the proportion of cells with activated caspase-3 in different cell cycle phases. Values shown in each quadrant of scattergrams represent the percentages of cells. Of note, the histograms also show the apoptotic subdiploid peak (sub-2N), especially enhanced in paclitaxel-treated NCI-H23 cells. (b) The effects of ReoT3D-paclitaxel combination on caspase-3 activation and DNA content were compared among NCI-H23, NCI-H460, EKVX and NCI-322M. These NSCLC cells were analyzed as described in (a). Shown are the percentages of cells positive or negative for activated caspase-3 in sub- G1/G1 or post-G1 phases. The data shown in (a) and (b) are representative of 2 experiments.

NCI-H23 cells were incubated with either ReoT3D (20 MOI) or paclitaxel (0.1 or 1 μM) alone or both in combination for 24 hours, after which non-adherent (detached) and adherent cells were harvested and evaluated for active caspase- 3 expression and DNA content. The total number of viable cells was obtained by the trypan blue dye exclusion method. Consistent with the caspase activity assay data (see above), incubation of NCI-H23 with ReoT3D resulted in an increased population of cells positive for cleaved active caspase-3 [22] as compared to untreated control (Figure 7a). DNA content analysis by propidium iodide (PI) staining demonstrated that the active caspase-3-expressing cells were equally distributed between G1 (2N DNA content) and G2/M phases (4N) after ReoT3D exposure (Figure 7a). In contrast, treatment with 0.1 or 1 μM paclitaxel led to marked increases in post-G1 cell populations, in particular cells with DNA content of 4N or greater (≥ 4N), and more than a third of these post-G1 cells with ≥ 4N DNA content were positive for activated caspase-3 (Figure 7a). In addition, paclitaxel treatment triggered an increase in the subdiploid (sub-2N) cell population, indicative of late apoptosis, in NCI-H23 cells (Figure 7a). The combination of ReoT3D and paclitaxel significantly decreased the number of viable cells (data not shown), while increasing the proportion of cells expressing active caspase-3, in particular in post-G1 phase, compared to ReoT3D- or paclitaxel-single treatment (Figure 7a).

Similar effects of ReoT3D-paclitaxel combination on cell cycle and apoptosis induction were also demonstrated in 3 other NSCLC cell lines, NCIH460, EKVX and NCI-H322M (Figure 7b). Paclitaxel treatment invariably increased the proportion of post-G1 cells in these cell lines, although the percentages of cells positive for activated caspase-3 were significantly smaller than in NCI-H23. The ReoT3D-paclitaxel combination consistently led to substantial increases in cells expressing active caspase-3, including in paclitaxel-resistant EKVX and NCI-H322M cells, and these increases appeared more prominent in post-G1 phase (Figure 7b). Indeed, statistical analysis demonstrated that the proportions of active caspase-3-positive cells were significantly different among the treatment groups in the post-G1 cell population (*P *< 0.005, one-way ANOVA), but not in the population with DNA content of 2N or less (sub-G1 and G1) (*P *= 0.05). In this subpopulation of post-G1 cells, the proportions of active caspase-3-expressing cells were significantly increased with the ReoT3D-paclitaxel combination treatment as compared to single therapy (*P *= 0.02 for both between-group comparisons, ReoT3D alone vs. ReoT3D+paclitaxel 0.1 μM, and paclitaxel 0.1 μM alone vs. ReoT3D+paclitaxel 0.1 μM; *P *= 0.02 and < 0.04 for between ReoT3D alone vs. ReoT3D+paclitaxel 1 μM, and paclitaxel 1 μM alone vs. ReoT3D+paclitaxel 1 μM, respectively). These data suggested that the combination of ReoT3D and paclitaxel enhanced apoptotic cell death, which was linked to cell cycle perturbation in NSCLC cells.

### Ultrastructural analysis of NSCLC cells treated with ReoT3D and paclitaxel

**Figure 8:**
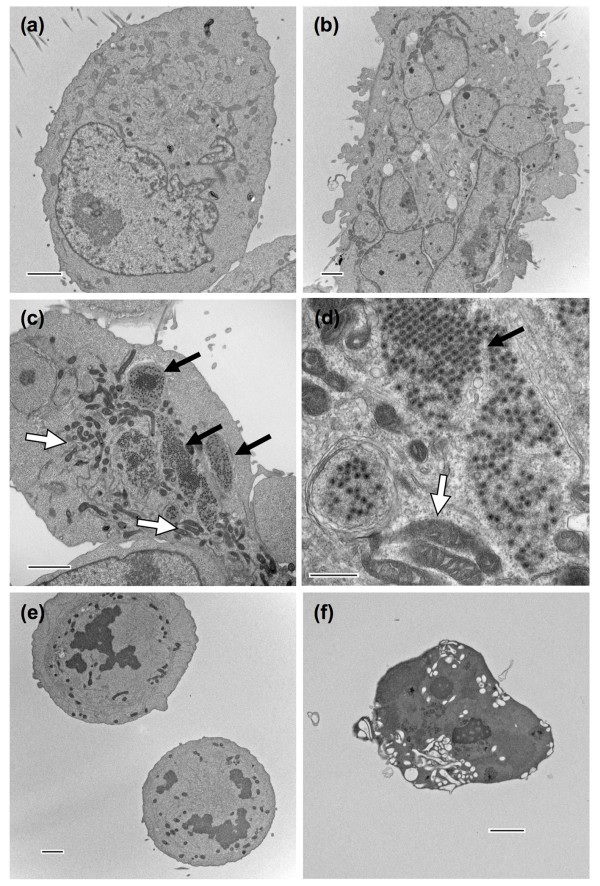
**Transmission electron micrographs of NCI-H23 cells treated with either paclitaxel (1 μM) or ReoT3D (MOI = 20) alone, or both in combination for 20 hours**. Compared to untreated control (a), the increased number of paclitaxel-treated NCI-H23 cells were found to be enlarged and multinucleated (b), whereas the cells infected with ReoT3D (c and d) contained numerous viral particles, mostly in viral inclusion bodies, which appeared globular in shape (black arrows). In addition, ReoT3D-infected cells appeared to contain an increased number of mitochondria (white arrows). When the cells were exposed to the combination of ReoT3D and paclitaxel, there were increased numbers of mitotic cells (e) as well as apoptotic cells characterized by condensed chromatin, cytoplasmic shrinkage and vacuolation (f). The results from survey of 100 cells for each treatment group are summarized in Table 3. Scale bars: 2 μm for (a), (b), (c), (e) and (f), and 500 nm for (d).

To further examine the relationship between cell cycle perturbation and apoptotic cell death induced by ReoT3D and paclitaxel, we examined the morphological changes of NCI-H23 cells treated with either ReoT3D or paclitaxel alone or in combination, using electron microscopy (EM). After overnight (~20 hours) treatment, the cells were directly fixed in situ and processed for the analysis. The EM study did not include dead cells that had detached before fixation. One hundred cells were surveyed within one 60-nm thin section for each treatment group to document significant changes as compared to untreated control (Table 3). When NCI-H23 cells were exposed to 1 μM paclitaxel overnight, an increased number of cells were found to be enlarged and multinucleated (Figure 8b), as verified by the survey (Table 3). These multinucleated cells, detected as post-G1 cells with ≥ 4N DNA content by flow cytometry (Figure 7a and 7b), represented the cells that had exited mitosis without cytokinesis. The cells infected with ReoT3D (MOI = 20) contained numerous viral particles, mostly in viral inclusion bodies that appeared globular in shape, and an increased number of mitochondria (Figure 8c and 8d). The combination of ReoT3D and paclitaxel resulted in notable increases in mitotic cells (Figure 8e), and apoptotic cells characterized by condensed and fragmented chromatin as well as cytoplasmic shrinkage and vacuolation (Figure 8f) (Table 3). Of note, although the number of viral particle-harboring cells that could be detected in the EM specimen was decreased with the ReoT3D-paclitaxel combination compared to ReoT3D alone (Table 3), the level of infectious progeny virion in the corresponding supernatant was higher than that of cells treated with ReoT3D alone (data not shown), indicating that the addition of paclitaxel enhanced reoviral production as discussed earlier.

**Table 3:** Summary of EM findings from survey of 100 adherent NCI-H23 cells

	**Cells with viral particles**		**Multinucleated cells**	**Nucleus**	
	**+**	**-**		**Condensed chromatin**	**Normal**
None	0	100	6	2	98
Paclitaxel 1 μM	0	100	22	11 (3*)	89
ReoT3D (MOI = 20)	37	63	3	4 (1*)	96
ReoT3D (MOI = 20) + Paclitaxel 1 μM	16	84	8	23 (17*)	77 (26^§^)

Note: NCI-H23 cells surveyed represent adherent cells that remained after exposure to the listed agents, alone or in combination, thus excluding detached dead cells. *Number of apoptotic cells characterized by condensed/fragmented chromatin, cytoplasmic shrinkage and vacuolation. ^§^Number of mitotic cells. Abbreviations: EM, electron microscopy; ReoT3D, reovirus type 3 Dearing; MOI, multiplicity of infection.

These data from flow cytometric and electron microscopic analyses strongly suggested that treatment with the combination of ReoT3D and paclitaxel caused prolonged mitotic arrest, which triggered accelerated apoptosis, resulting in synergistic cell killing in dually treated NSCLC cells.

## Discussion

Lung cancer is the leading cause of cancer mortality in both men and women in the United States [23] and all cancer deaths worldwide [24]. The most common form of lung cancer is NSCLC that includes squamous cell carcinoma, adenocarcinoma, and large cell carcinoma. Despite the tremendous efforts and progress in lung cancer research, treatment outcomes for non-localized NSCLC remain poor [25]. New treatment strategies are urgently needed to improve survival for advanced NSCLC patients. In the current study, we uncovered a potent oncolytic activity of ReoT3D against a panel of human NSCLC cell lines, in particular, NSCLC cell lines of adenocarcinoma or large cell carcinoma origin. The susceptibility of cancer cells to ReoT3D-mediated cytolysis has been attributed to increased Ras activity [11,12]. However, we did not observe any significant association between the ReoT3D-permissibility and the presence of Ras-activating gene mutations or activated Ras in human NSCLC cells. The lack of association has also been reported by others in human colon cancers [26]. It is possible that in addition to the activation status of Ras-associated pathways [11,12], there are other molecular determinants of ReoT3D-sensitivity, such as the cell surface density of putative ReoT3D receptors/coreceptors [27-29] and intracellular virion uncoating processes [30,31], all of which can affect ReoT3D infection efficiency.

The combination effects of herpesvirus or adenovirus-based oncolytic viral vectors and chemotherapeutic agents have previously been evaluated against different human cancers [32-37]. Synergistic activity was reported in the majority of these studies. However, combination regimens selected for the previous studies were mostly limited in scope in terms of dose range and the number of chemotherapeutic agents investigated. In the current study, we demonstrated that the oncolytic activity of ReoT3D against NSCLC cells could be significantly potentiated by a number of chemotherapeutic agents used in the treatment of NSCLC, including paclitaxel, cisplatin, gemcitabine and vinblastine. Combination analysis based on the Chou-Talalay's method [16,17] clearly showed significant levels of synergy between ReoT3D and each chemotherapeutic agent tested. Interestingly, we found that the drug sensitivity of each NSCLC cell line was an important determinant for the *in vitro *synergistic effect of ReoT3D-chemotherapeutic combination regimens, with the exception of ReoT3D-paclitaxel combination. It is conceivable that certain molecular changes conferring drug resistance can antagonize the process of ReoT3D-mediated cell killing. Our data, therefore, caution against the use of chemotherapeutic agents combined with ReoT3D for the treatment of NSCLC that have developed resistance to the agents. In contrast, the level of ReoT3D sensitivity did not appear to compromise the combination effects in NSCLC cells. Rather, the addition of chemotherapeutic agents may help accelerate ReoT3D-induced cell death process, which is otherwise slowed in NSCLC cells with low susceptibility to ReoT3D infection.

The most intriguing finding from our study was the synergistic effect of ReoT3D-paclitaxel combination consistently observed in all the NSCLC cell lines examined, regardless of the level of sensitivity to the compound. Because previous studies of oncolytic virus-chemotherapeutic combinations, in particular with paclitaxel, did not address the impact of drug resistance on the combination effects, we cannot ascertain whether our finding is unique to reovirus-containing combination therapy. Mammalian reoviruses are known to exploit microtubules for the formation of viral replication complexes (inclusion bodies) [38]. Based on our initial findings that the addition of paclitaxel to ReoT3D significantly increased the level of progeny virion production from all the NSCLC cell lines tested, we speculated that microtubule-stabilizing paclitaxel might have enhanced reoviral replication, resulting in a more efficient and synergistic oncolytic effect. However, the increased progeny virion production was not necessarily a unique outcome of ReoT3D-paclitaxel combination, but was also observed with ReoT3D-vinblastine combination in vinblastine-resistant NCI-H322M cells, where the combination of ReoT3D and vinblastine was found to be strongly antagonistic. Moreover, the addition of gemcitabine to ReoT3D treatment was not associated with an increased progeny virion production, regardless of the combination effects (synergy or antagonism) attained. These data suggested that the synergistic effect of ReoT3D and chemotherapeutic agents was not the direct result of enhanced lytic cell death, but more likely the manifestation of accelerated programmed cell death, which was triggered before virion assembly and release.

Reovirus has been shown to induce apoptotic cell death in a variety of cell types, including cancer cells [18,39]. Indeed, increased caspase activity and apoptotic cleavage of PARP were readily detectable in ReoT3D-treated NSCLC cells within 24 hours in the current study, as has been shown in ReoT3A-exposed cancer cells [18]. The combination treatment with ReoT3D and paclitaxel led to more robust caspase activation than ReoT3D alone with a significant leftward shift of the dose response curve in both paclitaxel-sensitive and -resistant NSCLC cell lines, suggesting that the enhanced apoptosis most likely constituted the synergistic cell killing by the combination, regardless of the level of paclitaxel sensitivity. To gain more insight into the mechanistic basis of accelerated apoptosis associated with the ReoT3D-paclitaxel combination, we examined the effects of ReoT3D and paclitaxel on caspase-3 activation in relation to cell cycle progression. While taxanes and other antimicrotubule agents are known to activate the spindle checkpoint and induce mitotic arrest [21], ReoT3D infection has been shown to effect cell cycle arrest at G1/S and G2/M [19,20]. Because arrests in cell cycle progression induced by anticancer agents are commonly followed by apoptosis [40], we hypothesized that these two agents with differing effects on cell cycle progression may have synergistically activated apoptotic pathways in dually treated NSCLC cells.

We found that the proportion of cells expressing activated caspase-3 was significantly increased by the ReoT3D-paclitaxel combination as compared to either ReoT3D or paclitaxel single treatment in each NSCLC cell line tested. Interestingly, the activation of caspase-3 was found more prominent in post-G1 cell population with ≥ 4N DNA content. Escape from mitotic arrest induced by spindle poisons such as taxanes and other antimicrotubule agents is commonly observed in cancer cells with impaired (weakened) mitotic checkpoint [21,41]. These cells that prematurely exit mitosis without proper cell division form large multinucleated cells with DNA content of 4N or greater, as demonstrated by EM in our study. After such mitotic slippage, some cells may undergo p53-dependent apoptosis, while others survive through senescence or continuing cell cycle (endoreduplication) [42], depending on the functions of p53, MAP kinase pathways, and p21-activated kinase [43,44]. In the current study, we found that NSCLC cells could efficiently escape from mitotic arrest induced by paclitaxel at 0.1 ~1 μM and survive at least for the first 24 hours of exposure, with the exception of NCI-H23 cells that were more prone to apoptosis upon paclitaxel exposure than 3 other NSCLC cell lines examined. While paclitaxel-treated NCIH460 cells ultimately underwent dramatic cell death after 48 hours, EKVX and NCI-H322M demonstrated considerable levels of resistance to paclitaxel-induced CPE. Nonetheless, the combination of ReoT3D and paclitaxel consistently accelerated the apoptotic process in post-G1 cells, including in paclitaxelresistant EKVX and NCI-H322M cells. This enhanced apoptosis appeared to have resulted from prolonged mitotic arrest, as corroborated by the EM data demonstrating that treatment with the ReoT3D-paclitaxel combination resulted in increased numbers of both mitotically arrested and apoptotic cells while decreasing the number of multinucleated cells as compared to paclitaxel alone.

The molecular mechanism of prolonged mitotic arrest induced by the ReoT3D-paclitaxel combination has yet to be elucidated. It is possible that ReoT3D infection may enhance the mitotic checkpoint activity in cancer cells with weakened mitotic checkpoint, for example, by upregulating the expression of mitotic checkpoint proteins (such as Mad1, Mad2, BubR1/Mad3, Bub1 and Bub3) [45], Cdk1 and/or cyclin B [45], or suppressing the anaphase-promoting complex/cyclosome activity [45]. Such ReoT3D-induced alterations in the mitotic regulatory network may reinforce the mitosis-arresting signal of taxanes, leading to prolonged mitotic arrest and apoptosis. Better understanding of the molecular consequences of ReoT3D infection on cell cycle checkpoint function and apoptotic signaling pathways in cancer cells will greatly enhance our ability to design rational combination therapies with proapoptotic oncolytic agent, ReoT3D, and various classes of anticancer agents. Concurrently, it is also of high importance to investigate potential consequences of ReoT3D-chemotherapeutic combinations on normal tissues in order to identify undesirable combination regimens that are associated not only with synergistic oncolytic activity, but also with enhanced toxicity in humans.

## Conclusion

The current study showed that the oncolytic activity of ReoT3D could be most effectively potentiated by taxanes through accelerated apoptosis, regardless of the level of taxane-sensitivity. Recently, preliminary findings from ongoing phase I clinical trials evaluating the safety of ReoT3D-taxane combination in patients with chemotherapy-refractory advanced tumors have demonstrated objective anticancer response in some patients without serious side effects [46,47], corroborating that ReoT3D-taxane combination regimens can achieve a durable oncolytic effect in clinical settings and thus should be further explored as a novel treatment modality for NSCLC.

## Methods

### Cell lines, virus and chemotherapeutic agents

Human NSCLC cell lines included in the NCI-60 cell line panel [48]: NCIH460 (large cell carcinoma), A549/ATCC (adenocarcinoma), HOP-62 (adenocarcinoma), NCI-H322M (bronchoalveolar carcinoma), NCI-H226 (squamous cell carcinoma), EKVX (adenocarcinoma), NCI-H23 (adenocarcinoma), NCI-H522 (adenocarcinoma), and HOP-92 (large cell carcinoma) were obtained from the In Vitro Cell Line Screening Program, DTP, NCI-Frederick. Of the 9 cell lines, NCI-H460, A549/ATCC, HOP-62, and NCIH23 have been found to have K-*ras *gene mutations [13]. L929 cells (mouse fibroblast L cells NCTC clone 929) were obtained from American Type Culture Collection (Manassas, VA). ReoT3D (REOLYSIN^®^) was provided by Oncolytics Biotech Inc. (Calgary, AB Canada). Cisplatin, paclitaxel, gemcitabine and vinblastine were obtained through the Drug Synthesis and Chemistry Branch, DTP, NCI.

### Determination of virus- and drug-induced cell death

ReoT3D- or drug-induced CPE was assessed by the XTT assay as described previously [49]. ED50 for ReoT3D was defined as the initial virus dose (MOI expressed in pfu/cell) that resulted in 50% cell viability at 48 hours post-inoculation as compared to untreated controls. Sensitivity of NSCLC cell lines to chemotherapeutic agents was expressed as the drug concentration that inhibited cell growth by 50% compared to untreated controls (IC50). Both ED50 and IC50 were calculated by using the software GraphPad Prism (GraphPad Software, Inc.). Cell viability was also assessed by intracellular ATP content (ATPLite™, PerkinElmer, Waltham, MA), when the level of caspases activity was evaluated in selected NSCLC cell lines treated with ReoT3D, paclitaxel or both agents in combination (see below).

### Evaluation of *in vitro *combination effects by the Chou-Talalay method

The combined effects of ReoT3D and each chemotherapeutic agent on cell survival were analyzed using the software CalcuSyn (Biosoft, Ferguson, MO), which applies the median-effect equation of Chou [16] and the CI equation of Chou and Talalay [17]. NCI-H460, HOP-92, NCI-H23, EKVX, NCI-H226 and NCI-H322M plated in 96-well microplates as above were exposed in triplicate to a serial dilution of each agent or both in combination using the constant ratio combination design [15] for 48 hours, followed by the XTT assay for cell viability determination. Calculated CIs were used to ascertain the presence of strong synergism (CI < 0.3), moderate synergism (0.3 < CI < 0.9), additive effect (CI = 1), antagonism (CI > 1) and strong antagonism (CI > 3.3) [15] between ReoT3D and chemotherapeutic agents.

### Reovirus plaque assay

Levels of infectious progeny virions in culture supernatants produced from ReoT3D-infected NSCLC cells were evaluated by plaque assay as previously described [50]. Infectious reovirus titers were expressed as pfu/mL of the original sample.

### Determination of activated Ras levels in NSCLC cells

Basal levels of activated Ras in 9 NSCLC cell lines were determined by Ras activation assay Biochem Kit (Cytoskeleton, Inc., Denver, CO) according to the manufacturer's instructions. Briefly, cell lysates were prepared from NSCLC cells using the kit lysis buffer, and aliquots of 2 mg protein were incubated with 20 μL of Raf1-RBD beads at 4°C for 1 hr, followed by centrifugation to pellet the Raf1-RBD beads. The pelleted beads were washed with wash buffer twice, and resuspended in 10 μL Laemmli sample buffer. The samples were separated by 12% Tris-glycine SDS-PAGE, and subjected to Western blot analysis with anti-Ras antibody (Cell Signaling Technology, Inc., Danvers, MA).

### Western blot analysis of PARP cleavage

NSCLC cells were cultured overnight in 6-well plates at a cell density of 2 × 10^6 ^cells/well, followed by incubation with ReoT3D, chemotherapeutic agents (gemcitabine, paclitaxel or vinblastine) or ReoT3D-chemotherapeutics in combination at indicated concentrations. Twenty-four to 30 hours after treatment, both adherent and non-adherent cells were collected and lysed in 150 μL cell lysis buffer (10 mM Tris/pH 7.4, 150 mM NaCl, 1 mM EDTA, 0.1% SDS, 1% sodium deoxycholate, 1% Triton X-100, 1 mM phenylmethylsulfonyl fluoride, 1 mM sodium orthovanadate, 50 mM sodium fluoride, 10 μg/mL aprotinin, 1 μg/mL leupeptin and 5 μM pepstatin). Cell lysates containing 20 μg protein were subjected to Western blot analysis with anti-PARP (Cell Signaling Technology, Inc.) and anti-β-actin (Cell Signaling Technology, Inc.) antibodies.

### Determination of caspase activity and ATP content by microplate-based assays

The levels of caspase activity and ATP content in NSCLC cells treated with ReoT3D alone or in combination with paclitaxel for 24 hours were evaluated by microplate-based assays, using a fluorometric pan-caspase assay (Homogeneous Caspases Assay; Roche Applied Science, Indianapolis, IN) and luminescence ATP detection assay (ATPLite™; PerkinElmer), respectively.

### Flow cytometric analysis of DNA content and caspase-3 activation

Adherent and non-adherent NSCLC cells harvested as above (see Western blot analysis) were fixed and permeabilized with BD Cytofix/Cytoperm™ solution (BD Biosciences, San Jose, CA) and stained with FITC-conjugated anti-active caspase-3 antibody (BD Biosciences), followed by incubation with PI/RNase staining buffer (BD Biosciences) for DNA content determination. The proportion of cells expressing active caspase-3 and DNA content were analyzed using a FACScan™ flow cytometer (BD Biosciences) as previously described [51].

### Transmission electron microscopy

NSCLC cells cultured in 6-well plates were processed in situ for electron microscopic analysis as previously described [52]. Thin sections (60 nm) were examined with a Hitachi H7000 transmission electron microscope.

### Statistical analysis

Results are reported as mean ± SD unless otherwise indicated. Statistical significance of differences was determined by one-way ANOVA or Student's t-test as appropriate. Differences were considered statistically significant when *P *< 0.05 (two-tailed).

## Competing interests

The authors, SS, JKM, QY, KN, REP, RHS and JET, declare that they have no competing interests. MCC is an employee of Oncolytics Biotech Inc.

## Authors' contributions

SS designed and directed the study, analyzed and interpreted all the data, and drafted the manuscript. JKM and QY carried out combination studies. JKM also performed Western blot analysis and caspase/ATP assays. QY carried out plaque assay and participated in flow cytometric analysis. KN performed TEM analysis. JET, MCC, RHS and REP participated in the study coordination, and helped draft the manuscript. All authors read and approved the manuscript.
